# Trends of mortality due to breast cancer in Poland, 2000–2016

**DOI:** 10.1186/s12889-020-8256-1

**Published:** 2020-01-29

**Authors:** Monika Burzyńska, Irena Maniecka-Bryła, Małgorzata Pikala

**Affiliations:** Epidemiology and Biostatistics Department, the Chair of Social and Preventive Medicine of the Medical University of Lodz, 90-752 Lodz, Poland

**Keywords:** Breast cancer, Neoplasms, Years of life lost, Mortality, Epidemiology, Trends, Poland

## Abstract

**Background:**

The aim of the study was to assess trends in mortality and the number of lost years of life due to breast cancer in the female population in the years 2000–2016, with consideration given to differences regarding the level of education and place of residence.

**Methods:**

The analysis was based on a database of the Central Statistical Office of Poland, containing information gathered from 92,154 death certificates of all Polish female inhabitants who died in the period 2000–2016 due to breast cancer. The SEYLL_p_ (Standard Expected Years of Life Lost per living person), the SEYLL_d_ (per deaths), the APC (Annual Percentage Change), the AAPC (Average Annual Percentage Change) were calculated to determine years of life lost.

**Results:**

The mean age of women who died from breast cancer increased in the study period from 64.7 years to 69.7. The SEYLL_p_ index (per 100,000) increased to 776.8 years in 2016 (AAPC = 0.5%). The most unfavorable changes were observed in the group of women with secondary education. In 2004, the SEYLL_p_ values started to grow at a rate of 2.3% and since 2011, they have been higher than amongst women with elementary education. In the years 2000–2016, the authors observed that SEYLL_p_ was steadily declining (APC = -1.0%) in the group of inhabitants of rural areas, whereas with regards to city dwellers, the SEYLL_p_ index has been increasing since 2004 (APC = 0.5%), which has resulted in increased disproportions regarding the place of residence.

**Conclusions:**

The results of this study showed that breast cancer is becoming a serious epidemiological problem in Poland. There is the need to intensify activities among women at highest risk group and it should be the starting point for making key decision in combating breast cancer.

## Background

A review of epidemiological data on morbidity and mortality due to cancer indicates that estimates of the global cancer burden has increased to 18,1 million cases and 9,6 million deaths were recorded in 2018 [1]. The International Agency for Research on Cancer estimates that one in five men and one in six women worldwide will develop cancer in their lifetime, while one in eight men and one in 11 women will die of this disease. Many factors are responsible for this increase; particularly, the advancing age of the global population and increased exposure to cancer risk factors associated with social and economic development. Data from 2018 also indicate that cancer incidence is 2–3 times higher in countries with high Human Development Index (HDI) than in countries with low or medium value of this index [1]. Breast cancer is the most common kind of cancer among women. It is the second most common cancer which affects the worldwide population. In 2018, 2,088,849 new cases of the disease were detected and this number constituted 11.6% of all cancers diagnosed at that time. Deaths due to this reason constituted 6.6% of the total number of cancer deaths. It should be mentioned that significant territorial differences were observed in this respect. The highest standardised death rates for breast cancer were reported in Belgium (113.2 per 100,000) and in Luxembourg (109.3 per 100,000) [1]. In 2018, breast cancer accounted for 10.9% of all diagnosed cancers, with an incidence rate of 59.1 per 100,000 and was the second most common kind of cancer in the global population. Deaths due to this reason constituted 6.1% of all cancer deaths (the third cause of deaths in this class) [2].

Between the mid-1970s and the middle of the first decade of the twenty-first century, breast cancer was the most common cause of mortality of all cancer-related deaths, affecting the female population in Poland. Since 2007, it has been the second cause of death in the group of cancer-related deaths, which is mainly caused by the increase in morbidity and mortality due to lung cancer [3].

Breast cancer is a multifactorial disease. Scientific studies confirm that there are numerous risk factors which trigger neoplastic processes in the breast region. The most important are: changes in the procreative pattern, obesity, lack of physical activity, genetic predisposition and age [4, 5].

Due to the growing burden of cancer in European countries, it is highly important to take any actions which would make the fight against cancer more effective. For this reason, the World Health Organization and Union for International Cancer Control recommend popularization of primary and secondary prevention and systematic implementation of modern technologies in diagnostics, treatment and rehabilitation. Many countries have introduced programmes related to reducing the economic and social consequences of breast cancer. Screening programmes appeared to be effective tools because once they were introduced, mortality rates declined by 20–30% [6]. The effectiveness of the above mentioned activities can be assessed only with the use of scientific methods, such as: analyses of incidence rates, mortality rates, the population survival rate or the number of years of life lost due to cancer death. Results of these analyses are reflected in strategies of combating cancer in many European countries [7]. There are some studies on incidence and mortality due to breast cancer in Poland however our study is the first covering life years lost due to this cause. The aim of this study was to assess trends in mortality and mortality-related lost years of life due to breast cancer in the female population, in the years 2000–2016, with consideration given to differences regarding the level of education and place of residence (urban areas – rural areas).

## Methods

The research project was granted an approval of the Bioethics Committee of the Medical University of Lodz on 22 May 2012 No. RNN/422/12/KB.

The analysis was based on a database of the Central Statistical Office of Poland, containing information gathered from 6,384,495 death certificates of all inhabitants of Poland who died between 2000 and 2016. Of all deaths in Poland in studied period, 92,154 women died of breast cancer (according to the ICD-10 – International Statistical Classification of Diseases and Health Related Problems – Tenth Revision – coded as C50). The crude death rates (CDR) and standardized death rates (SDR) were calculated by the authors. The direct method of the standardization procedure was performed, in compliance with the European Standard Population, updated in 2012 [8].

Years of life lost were counted and analysed by the method described in Global Burden of Disease (GBD) [9]. The SEYLL index (Standard Expected Years of Life Lost) was used to calculate the number of years of life lost by the studied population in comparison to the years lost by the referential (standard) population [10]. The authors of this manuscript have used this method in their other studies on lost years of life due to cancers. Further details on the SEYLL index are available in the authors’ earlier publications [11, 12].

The SEYLL per person (SEYLL_p_) index was used, which is a ratio of SEYLL and the size of the population, calculated per 100,000 inhabitants. The authors also applied the SEYLL per death (SEYLL_d_) index, being a ratio of SEYLL and the number of deaths due to a particular cause; that is, it expresses the number of YLL calculated per one dead person [13].

The joinpoint models and *Joinpoint Regression* program, a statistical software package developed by the U.S. National Cancer Institute for the Surveillance, Epidemiology and End Results Program [14] were used to analyze the time trends of number of death, CDR and SEYLL. This method is an advanced version of linear regression, where time trend is expressed with a broken line, being a sequence of segments joined in joinpoints. In these points, the change of the value is statistically significant (*p* < 0.05). The annual percentage change (APC) of CDR, SDR, SEYLL_p_, and SEYLL_d_, for each segment of broken lines and average annual percentage change (AAPC) for a full range of analysed years with corresponding 95% confidence intervals (CI) were calculated also.

In order to compare the number of years of life lost by women due to breast cancer in particular categories, such as place of residence (urban area/rural area), education level, the authors calculated Rate Ratio (RR), being a ratio of SEYLL_p_ in less privileged groups and SEYLL_p_ in more privileged groups with corresponding 95% confidence intervals (CI) [15].

## Results

The number of deaths due to this reason was steadily increasing. In 2000, 4749 deaths occurred, whereas in 2016, the number was 6576. Crude death rates (CDR) were 24.1 in the year 2000 and 33.1 in the year 2016 (per 100,000 female population) (Table 1). In the analysed study period, the average annual percentage change (AAPC) was 2.0% (*p* < 0.05). The annual percentage change (APC) increased from 1.2% in 2000–2012 up to 4.2% in 2012–2016 (*p* < 0.05) (Table 2).

**Table 1:** Number of deaths and values of crude death rate (CDR), standardized death rates (SDR), standard expected years of life lost (SEYLL), standard expected years of life lost per living person (SEYLL_p_), and standard expected years of life lost per death (SEYLL_d_) due to breast cancer in Poland in 2000–2016

Year	Number of deaths	CDR (per 100,000)	SDR (per 100,000)	SEYLL	SEYLL_p_ (per 100,000)	SEYLL_d_ (per deaths)
2000	4749	24.1	30.4	114,967	711.6	24.2
2001	4876	24.7	30.8	116,177	713.5	23.8
2002	4880	24.8	30.3	116,948	713.5	24.0
2003	4983	25.3	30.5	117,534	712.6	23.6
2004	4938	25.1	29.7	116,138	699.9	23.5
2005	5163	26.2	30.7	118,491	710.1	22.9
2006	5255	26.7	30.6	119,496	712.8	22.7
2007	5300	26.9	30.2	119,204	708.3	22.5
2008	5399	27.4	30.3	120,947	716.4	22.4
2009	5311	26.9	29.3	117,744	695.7	22.2
2010	5285	26.6	28.7	116,056	681.7	22.0
2011	5497	27.7	29.3	121,122	710.4	22.0
2012	5651	28.4	29.6	122,116	715.7	21.6
2013	5881	29.6	30.3	126,636	742.5	21.5
2014	6024	30.3	30.7	128,947	756.2	21.4
2015	6386	32.2	31.9	129,348	759.2	20.3
2016	6576	33.1	32.5	132,279	776.8	20.1

Source: own calculations

**Table 2:** Time trends of CDR, SDR, SEYLL_p_, and SEYLL_d_ due to breast cancer in Poland in 2000–2016—joinpoint regression analysis

	Number of joinpoints	Years	APC (95% CI)	AAPC (95% CI)
CDR	1	2000–2012	1.2* (0.9; 1.5)	2.0* (1.6; 2.4)
		2012–2016	4.2* (2.6; 5.8)	
SDR	1	2000–2012	-0.4* (−0.7; − 0.1)	0.4* (0.1; 0.7)
		2012–2016	2.6* (1.1; 4.2)	
SEYLL_p_	1	2000–2010	-0.3* (− 0.5; − 0.1)	0.5* (0.3; 0.7)
		2010–2016	1.8* (1.4; 2.3)	
SEYLL_d_	1	2000–2014	−0.9* (− 1.0; − 0.8)	−1.2* (− 1.4; − 1.0)
		2014–2016	− 3.0* (− 5.0; − 1.1)	
SEYLL_p_ according to level of education				
high	2	2000–2004	−9.4* (− 12.9; −5.7)	−2.4* (− 3.9; − 1.0)
		2004–2013	− 1.5* (− 2.8; − 0.1)	
		2013–2016	4.7 (− 1.6; 11.5)	
secondary	1	2000–2004	−2.3 (− 5.4; 0.0)	1.1* (0.3; 1.9)
		2004–2016	2.3* (1.6; 2.9)	
elementary	1	2000–2002	−9.9* (− 18.6; − 0.2)	− 1.5* (− 2.7; − 0.3)
		2002–2016	− 0.2 (− 0.7; 0.2)	
SEYLL_p_ according to place of residence				
urban	1	2000–2004	−4.6* (−8.8; − 0.3)	−0.1 (− 1.2; 1.1)
		2004–2016	0.5* (0.6; 2.3)	
rural	0	2000–2016	−1.0* (− 1.6; −0.3)	

Source: own calculations

*p < 0.05

Standardized death rates were decreasing in the years 2000–2012 (APC = − 0.4%, p < 0.05); after 2012, they started to increase at an average annual rate of 2.6% (p < 0.05) (Table 2). As a result of these changes, SDR which was 30.4 per 100,000 females in the year 2000 increased to 32.5 in the year 2016 (Table 1).

The increasing values of death rates contributed to an increase in the number of standard expected years of life lost (SEYLL). In 2000, the SEYLL value was 114,967 and in 2016, it was 132,279. The SEYLL_p_ index increased from 711.6 in 2000 to 776.8 in 2016 (per 100,000 female population) (Table 1). In the whole analysed period, AAPC was 0.5% (*p* < 0.05); however, after a small but statistically significant decrease in 2000–2010 (APC = -0.3%), SEYLL_p_ values started to increase from 2011 at an annual rate of 1.8% (p < 0.05) (Table 2).

**Fig. 1:**
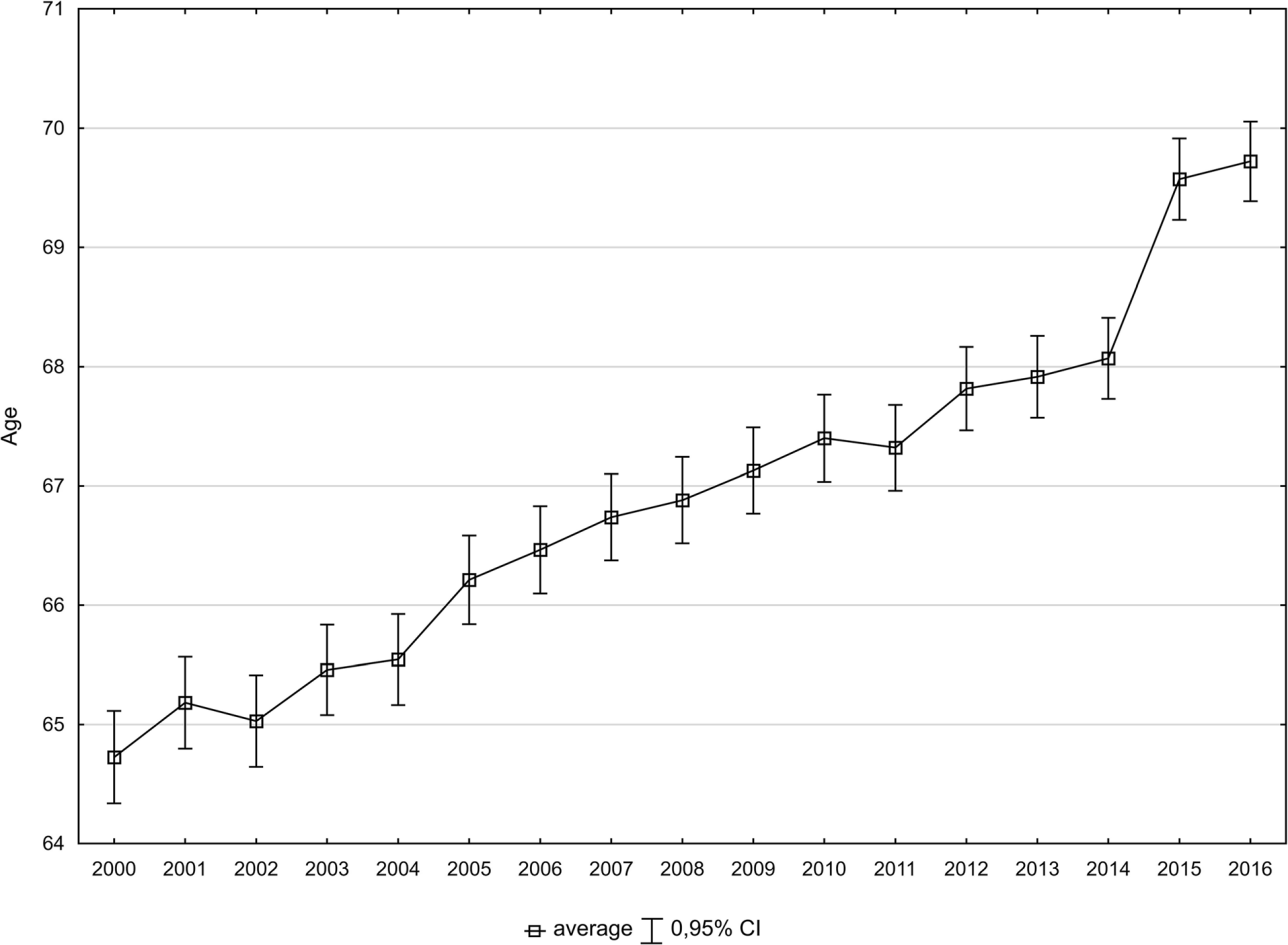
The average age of women who died of breast cancer, Poland, 2000–2016. Source: own calculations

The average age of women who died of breast cancer was steadily increasing. In 2000, it was 64.7 years, while in 2016, it was 69.7 years (Fig. 1). This increase contributed to a decreased number of lost years of life, calculated per the number of women who died due to breast cancer. The SEYLL_d_ index was 24.2 in the year 2000 and 20.1 in the year 2016. AAPC for the period 2000–2016 was − 1.2 (*p* < 0.05) and the rate of the decline increased from − 0.9% in 2000–2014 (p < 0.05) to − 3.0% in 2014–2016 (p < 0.05) (Table 2).

**Fig. 2:**
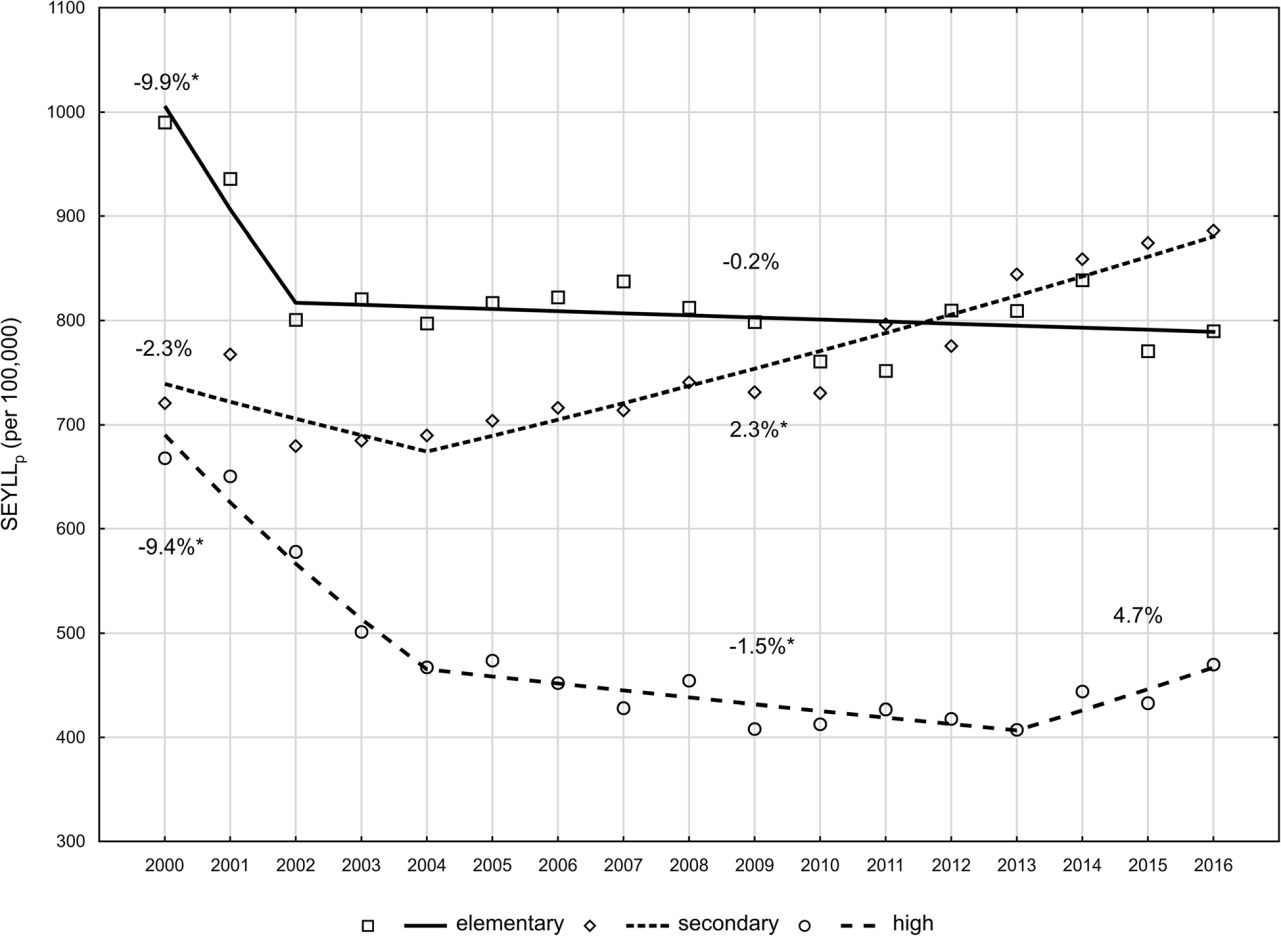
Time trends of the SEYLL_p_ index by aducation in 2000–2016 in Poland. *Source: own calculations*

An analysis of trends in this area indicates, however, that after a rapid decline at a rate of − 9.4% (p < 0.05) in the period 2000–2004, the rate of decline decreased to − 1.5% (p < 0.05) in 2004–2013. After 2013, SEYLL_p_ indices started to increase at an annual average rate of 4.7% (*p* > 0.05) (Fig. 2) The authors observed that the number of standard expected years of life lost depended on the level of education. The lowest values of SEYLL_p_ indices were noted in the group of women with university education (667.8 in 2000 and 469.8 in 2016 per 100,000) (Table 3). The highest values of SEYLL_p_ indices for 2000 were observed in the group of women with elementary education (990.1 per 100,000) (Table 3). In this educational group, the authors observed a decrease in SEYLL_p_ indices in 2000–2002 (APC = -9.9%, *p* < 0.05) which stabilised in the period 2002–2016 (APC = -0.2, *p* > 0.05) (Fig. 2) In 2016, the SEYLL_p_ value, calculated in the group of women with elementary education, decreased to 789.7 per 100,000. The Rates Ratio (RR), being a ratio of SEYLL_p_ in the group of women with elementary education to SEYLL_p_ in the group of women with university education, increased from 1.08 in 2000 to 1.89 in 2016 (Table 3). The most unfavourable changes were observed in the group of women with secondary education. In 2000, SEYLL_p_ was 720.9 per 100,000 and was lower than in the group of women with elementary education. From the year 2004 onwards, the SEYLL_p_ values were increasing at a rate of 2.3% (*p* < 0.05). As a consequence, since 2011, SEYLL_p_ values in the group of women with secondary education have been higher than in women with elementary education. RR for women with secondary and university education was 1.48 in 2000 and 1.68 in 2016.

**Table 3:** Years of life lost (SEYLL_p_) due to breast cancer and Rate Ratio (RR) according to level of education and place of residence, 2000 and 2016, Poland

	SEYLL_p_		RR (95% CI)	
	2000	2016	2000	2016
Educational level				
high (ref)	667.8	469.8	1.00	1.00
secondary	720.9	886.1	1.08 (1.06; 1.10)	1.89 (1.86; 1.92)
elementary	990.1	789.7	1.48 (1.45; 1.52)	1.68 (1.65; 1.71)
Place of residence				
rural (ref)	814.1	688.6	1.00	1.00
urban	812.9	830.6	1.00 (0.99; 1.01)	1.21 (1.19; 1.22)

Source: own calculations

**Fig. 3:**
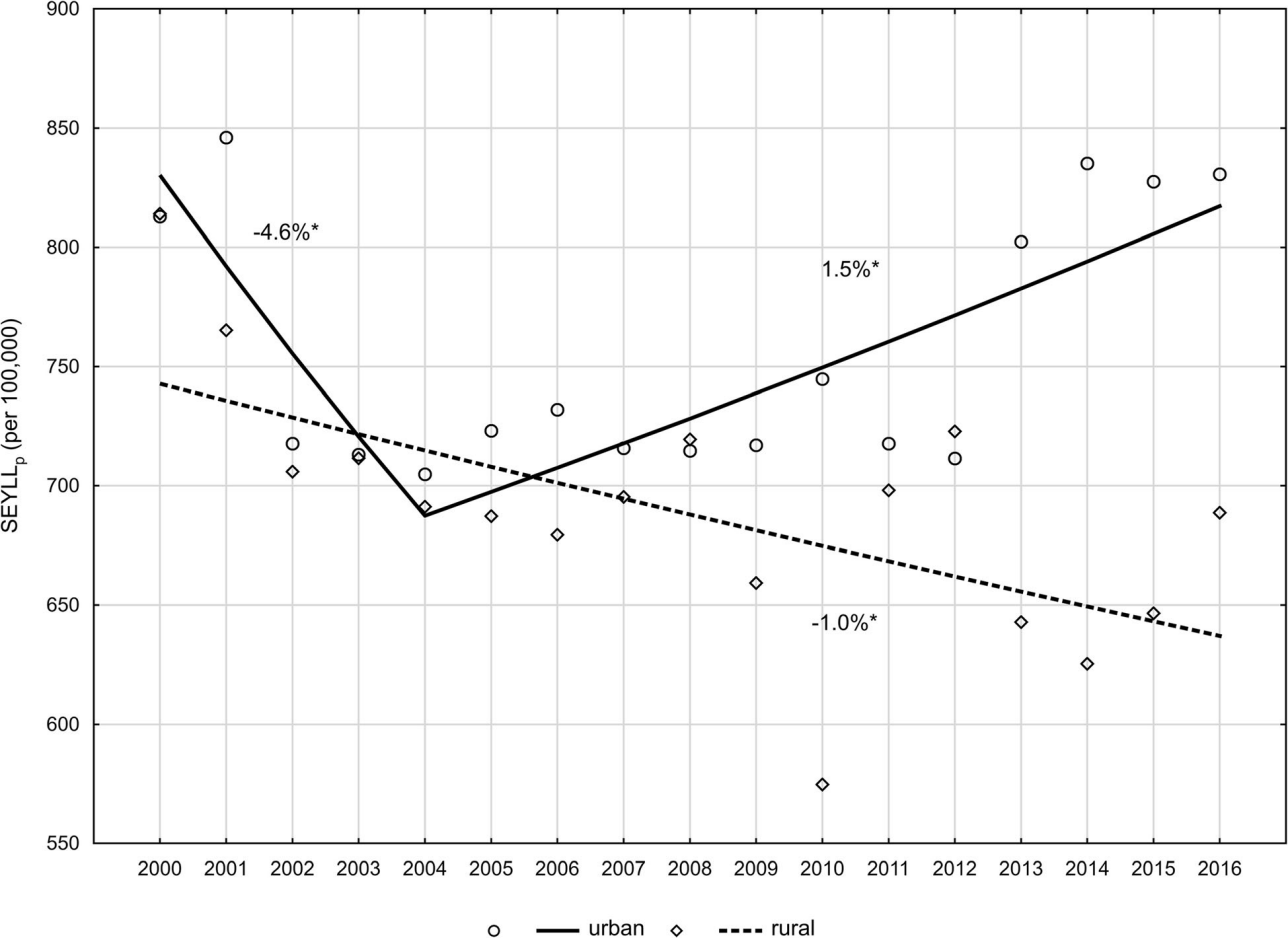
Time trends of the SEYLLp index by place of residence in 2000–2016 in Poland. Source: own calculations

Differences in the number of lost years of life due to breast cancer between female inhabitants of urban and rural areas were also subject to an analysis. In 2000, the differences were hardly noticeable (814.1 vs. 819.9 per 100,000). Change trends in the period 2000–2016 in both the compared groups, however, differed in terms of the rate and direction. In the group of women inhabiting rural areas, the authors observed a steady declining trend (APC = -1.0%, *p* < 0.05). In the period 2000–2004, SEYLL_p_ values decreased at a rate of − 4.6% (p < 0.05) in inhabitants of rural areas. In the year 2004, they started to increase at an annual rate of 1.5% (p < 0.05) (Fig. 3). As a result of these changes, the urban-rural RR increased from 1.00 in 2000 to 1.21 in 2016.

## Discussion

Favourable trends of mortality due to breast cancer were recorded in 2002–2012 in EU countries. The standardised mortality rate decreased in this period from 17.9 to 15.2 per 100,000 (world standard). A further decline, up to 13.4, is expected to occur by 2020 [16]. Despite the fact that Poland is a member of the EU and is one of the high-income countries, results of this study (Table 1) showed that since 2012, breast cancer-related mortality rates have been increasing.

In order to explain these unfavourable trends in Poland, we should point out changes in the age structure of women. Data from the National Cancer Registry indicate that most breast cancer-related deaths occur after the age of 50 (90%) [17]. The percentage of women over 50 years old in Poland in 2000 was 30.7% and it rose to 39.4% in 2016 [18]. Crude death rates started increasing in 2012 at an average annual rate of 4.2%. In the same period, standardised death rates were also increasing. However, the rate was lower, i.e. 2.6%. This fact indicates that changes in the age structure are not the only cause of unfavourable trends.

Population screening tests undoubtedly reduce mortality from breast cancer. Due to the identification of cancer at earlier stages, treatment is more likely to succeed. In Poland, a population screening programme for breast cancer started as late as in 2006. In contrast, in the United States, such tests had been introduced 20 years before. Unfortunately, no epidemiological changes, related to female participation in this programme, have been noticed yet. It should also be stressed that this programme will not appear beneficial for the entire population if participation rates remain that low. In 2010, 40% of women participated in the programme [19]. However, indirect effects of screening tests can be observed. The highest incidence is observed amongst 50–69 year-old women who underwent screening tests [20]. Introduction of screening tests also affected the stage of cancer at the time of diagnosis. We observe that the number of in situ and advanced neoplasms was steadily growing, whereas the number of regionally advanced neoplasms is steadily decreasing. The simplest method of assessing the effectiveness of prophylaxis programmes is to compare incidence rates before and after implementation of those programmes, with particular consideration given to the advancement of level of the neoplasm. For effective prophylaxis programmes, we can expect an increased incidence rate of early-stage cancers and a decreased incidence rate of cancers at later stages. Prophylaxis programmes which were implemented in Lithuania [21], Germany [22], Italy [23] as well as Iceland [24] and East Anglia [25] proved satisfactorily effective. The effectiveness of screening programmes started to be assessed after 30 years of observation; the 10 years observation period in Poland therefore precludes any assessment of efficiency or effectiveness regarding to mortality due to breast cancer [26].

A relative survival rate amongst cancer patients in a given period is one of the best measures which enables researchers to compare oncology care systems in particular countries. It is assumed that 1-year survival rate is an indication of effectiveness of early detection and diagnostic programmes, while higher 5-year survival rates show quality of clinical care and treatment effectiveness [27]. In Poland, in the years 2000–2002, 1-year survival rate for women diagnosed with breast cancer was 92.8%, while in the period 2003–2005, the rate was 93.2%. 5-year survival in breast cancer patients, during the first decade of the twenty-first century, slightly increased, i.e. from 75.0% in 2000–2002 to 77.2% in 2003–2005 [28]. The absolute number SEYLL and the SEYLL_p_ index values increased in 2000–2016, which is associated with an increasing number of deaths due to breast cancer. On the other hand, the values of SEYLL_d_ indices per one woman who died of breast cancer decreased. In 2000, the SEYLL_d_ index due to breast cancer was 24.2, whereas in 2016, it was 20.1 which is caused by a higher mean age of women with breast cancer and higher rates of 5-year survival. It should be noted, however, that survival rates of women with breast cancer in Poland are still much lower than those observed in other European countries. With regards to 5-year survival of women affected by breast cancer, Poland occupies one of the last places in Europe and is far behind countries such as France and Germany [29]. In Poland, in comparison to average European statistics, 1-year survival rates were over 4 percentage points lower, while 5-year survival rates were lower by more than 10 percentage points. There are many reasons for this situation, but what is striking is the fact that far too many cancer cases are diagnosed too late, thus making the prognosis very poor. Other factors determining differences also include: availability of oncological care and frequency of screening test [30].

The growing number of deaths from breast cancer in Poland contributes to a growing number of lost years of life. In the years 2010–2016, SEYLL_p_ indices were increasing at an annual rate of 1.8%. In contrast, SEYLL_d_ indices were decreasing in that period. Each woman who died of breast cancer in Poland in 2000 lost on average 24.2 years and over 4 years less (20.1) if she died in the year 2016. The reason for that difference was the fact that the mean age at death from breast cancer in the years 2000–2016 changed from 64.7 to 69.7 years. Besides, the aforementioned survival rates also improved. An analysis of 20 diseases, being the greatest contributors of mortality in Poland in 2011, reveals that breast cancer occupied the 7th place in the group of women in terms of the value of the SEYLL_d_ index [31].

This study also focused on differences in mortality due to breast cancer in subjects with different education levels. The lowest SEYLL_p_ values occur in the group of women with university education; however, we observed that after years of decline, the indices started to increase in this educational group in around the year 2013 at a fast annual rate of 4.7%. RR in the group of women with elementary education vs. university education was 1.7 in 2016. In contrast, for cervical cancer, RR was 5.8 [11]. This confirms the observation, noted in other studies conducted both in Poland [32] and in 22 European countries [33], that breast cancer is the most egalitarian of all cancers.

Our study also revealed differences in the number of lost years of life between inhabitants of urban and rural areas. In 2000, values ​​of indices in the city and countryside were almost equal. In the years 2000–2016, SEYLL_p_ values were steadily declining in inhabitants of rural areas, whereas in the group of city inhabitants, we have been observing a growing trend since 2004, which indicates disproportions regarding the place of residence even greater. Similar results were obtained in a study conducted in one of Polish voivodeships (świętokrzyskie voivodeship) in the years 2002–2013 [34]. Studies conducted in 1995–2013 in North America showed a similar relationship. Incidence rates, calculated in the analysed period, were higher in urban areas than in rural areas [35, 36]. Reasons for these differences include: increased exposure to carcinogenic factors in cities, different city lifestyle, as well as higher fertility rates in women inhabiting rural areas, which reduces the risk of the disease.

## Conclusions

Rising mortality trends and an increase in the number of lost years of life imply that breast cancer is becoming a serious epidemiological problem in Poland. The results of this study revealed noticeable inequality in mortality due to breast cancer regarding to educational level and place of residence. Therefore, it is necessary to enhance activities to encourage female participation in screening mammography, aimed primarily at the most vulnerable population groups i.e. with elementary education and inhabitants of urban areas. It should be the starting point for making key decision in combating breast cancer by public health institutions for disease control and prevention. There is also a need to continue monitoring changes in this area.

## Data Availability

The datasets used and/or analysed during the current study are available from the corresponding author on reasonable request.

## Abbreviations

AAPC: Average annual percentage change
APC: Annual percentage change
CDR: Crude death rate
CI: Confidence interval
EU: European Union
GBD: Global burden of disease
HDI: Human development index
ICD-10: International Statistical Classification of Diseases and Health Related Problems – Tenth Revision
RR: Rate ratio
SDR: Standarized death rate
SEYLL: Standard expected years of life lost
SEYLL_d_: Standard expected years of life lost per death
SEYLL_p_: Standard expected years of life lost per living person
YLL: Years life lost
